# Assessing completeness of patient medical records of surgical and obstetric patients in Northern Tanzania

**DOI:** 10.1080/16549716.2020.1765526

**Published:** 2020-06-01

**Authors:** William Lodge, Gopal Menon, Salome Kuchukhidze, Desmond T. Jumbam, Sarah Maongezi, Shehnaz Alidina, Boniface Nguhuni, Ntuli A. Kapologwe, John Varallo

**Affiliations:** a Department of Global Health and Social Medicine, Harvard Medical School, Boston, MA, USA; b Department of Adult Non-communicable Diseases, Ministry of Health, Community Development, Gender, Elderly, and Children (Mohcdgec), Tanzania, Dodoma; c Department of Health, Social Welfare and Nutritional Service, President’s Office - Regional Administration and Local Government, Dodoma, Tanzania; d Safe Surgery 2020 Project, Jhpiego, Baltimore, MD, USA

**Keywords:** Data quality, surgical care, Tanzania, surgical site infections, sepsis

## Abstract

**Background:**

Strengthening surgical services in resource-constrained settings is contingent on using high-quality data to inform decision making at clinical, facility, and policy levels. However, the evidence is sparse on gaps in paper-based medical record quality for surgical and obstetric patients in low-resource settings.

**Objective:**

We aim to examine surgical and obstetric patient medical record data quality in health facilities as part of a surgical system strengthening initiative in northern Tanzania.

**Methods:**

To measure the incidence of Surgical Site Infections (SSIs), sepsis and maternal sepsis surgical and obstetric inpatients were followed prospectively, over three months in ten primary, district, and regional health facilities in northern Tanzania. Between April 22nd to May 1st, 2018, we retrospectively reviewed paper-based medical records of surgical and obstetric patients diagnosed with SSIs, post-operative sepsis, and maternal sepsis in the three-month follow-up period. A data quality assessment tool with18 data elements related to documentation of SSIs and sepsis diagnosis, their respective symptoms and vital signs, inpatient daily monitoring indicators, and demographic information was developed and used to assess the completeness of patient medical records.

**Results:**

Among the 157 patients diagnosed with SSI and sepsis, we found and reviewed 68% of all medical records. Among records reviewed, approximately one third (34%) and one quarter (23%) included documentation of SSI and sepsis diagnoses, respectively. 6% of reviewed records included documentation of all SSI and sepsis diagnoses, symptoms and vital signs, inpatient daily monitoring indicators, and demographic data.

**Conclusions:**

Strengthening data quality and record-keeping is essential for surgical team communication, continuity of care, and patient safety, especially in low resource settings where paper-based records are the primary means of data collection. High-quality primary health information provides facilities with actionable data for improving surgical and obstetric care quality at the facility level.

## Background

The Lancet Commission has primarily driven the growing global movement to expand access to essential and emergency surgical and anesthesia care on Global Surgery report in 2015, which identified a gap of 143 million surgical procedures in low-income and middle-income countries (LMICs) to meet the need [1]. This movement, along with others, has brought the importance of surgical quality to the forefront of global health priorities [2,3]. To address the high burden of conditions amenable to surgical care in Tanzania, the Ministry of Health, Community Development, Gender, Elderly, and Children launched its first National Surgical, Obstetric, and Anesthesia Plan in 2018 [4,5]. However, the evidence on improving surgical quality and generating scientific evidence through high-quality data is sparse. Such improvement is contingent upon the availability of reliable data to assess the current status of surgical care quality, evaluate the need, identify the gaps and thus inform priorities both at the facility and national policy levels [6,7]. Effective health quality improvement depends on the complete, accurate, and timely flow of data between primary, secondary, and regional health facilities and the central data repository [8]. At the facility level, having access to reliable and complete patient data is essential for surgical and obstetric providers as well as hospital managers to coordinate care, identify areas for improvement and ensure effective and efficient delivery of safe, high-quality care [9]. While there has been an increase in the availability and utilization of electronic medical records in sub-Saharan Africa over the last decade, there are significant gaps in the infrastructure to support the use of electronic records in LMICs [10]. Since paper-based record-keeping is still the primary medical data collection method in LMICs, the existing paper records must be better utilized to ensure patient safety and high-quality surgical and obstetric patient care.

Tanzania has a two and a half decade history of using a Health Management Information System (HMIS), also known as Arrifa Aa Uendeshji Huduma za Afya (MTUHA), a paper-based data entry system of 12 HMIS booklets [11]. These booklets are meant to cover all health services and are mandatory to be completed and reported to the district health authority through a national system. Their focus is primarily on maternal and child health indicators. Surgical care indicators, such as rates of surgical site infections (SSIs), post-operative sepsis, and maternal sepsis, which are often used as measures of surgical quality, are not routinely reported [11,12]. Due to the absence of mandatory national reporting for these indicators, patient medical data are often poorly recorded and underutilized, which creates barriers for peri-operative monitoring, continuity of care, and patient safety [11,13]. Medical records for surgical patients, which are separate from the MTUHA system, help bridge this gap. Even though medical records for surgical and obstetric patients vary by facility, they typically include essential elements such as patient history, a partograph for obstetric patients, post-operative notes, and a drug administration chart. Medical records are used as the primary means of documentation of perioperative patient clinical information, and communication between the surgical and ward teams. The importance of medical records in the continuity of perioperative patient care warrants a thorough exploration of the current gaps in data recording to identify areas of improvement, although the authors found no published literature on the state of surgical data collection in Tanzania

To better understand the surgical data quality, we examined the completeness of surgical and obstetric patient medical records in 10 health facilities in northern Tanzania, focusing on the essential components needed for perioperative patient monitoring. To our knowledge, this is the first quantitative assessment of medical record completeness in primary, district, and regional facilities in Tanzania.

## Methods

### Identification of SSI and sepsis patients

Our data quality assessment was conducted as part of a longitudinal, multi-centered, quasi-experimental study to examine the impact of a multi-component intervention in ten intervention and ten control facilities in Tanzania’s Lake Zone region. The study protocol is published elsewhere [14]. Briefly, the study included 4 health centers, 11 district hospitals, and 5 regional referral hospitals. On average, 82 surgical procedures were conducted monthly per facility, which was mostly cesarean sections.

The baseline study was conducted between February 1st, 2018, and May 1st, 2018, by recently graduated Tanzanian medical doctors who had one or two years of specialized training in clinical and/or surgical care and were trained as data collectors. The data collectors followed surgical inpatients prospectively using a screening tool to diagnose SSI, post-operative sepsis, and maternal sepsis for the duration of the patient’s stay up to 30 days. All patients who were over the age of 5, had undergone surgery in a major operating room or delivered at the facility vaginally or through a cesarean section were included in the study. All minor surgeries, visiting surgeons’ patients, antenatal patients, women with spontaneous abortions, and surgical outpatients were excluded. The list of minor surgeries excluded from the study is listed in Appendix I. To make the diagnosis, data collectors conducted daily patient rounds independently of the surgical teams and reviewed patient charts. When information could not be obtained through clinical examination or chart review, data collectors discussed cases with surgical providers.

The research team developed the SSI screening tool based on the CDC criteria for diagnosing and classifying SSI [15]. Both the post-operative and maternal sepsis screening tools were adapted for low-resource settings from Surviving Sepsis Campaign guidelines, which was based on Second International Consensus on Sepsis [16–18]. The tools were pre-tested by the research team and the data collectors at two secondary health facilities in Dar es Salaam. To ensure consistency in interpretation, the research team and data collectors debriefed the process and identified inconsistencies, unclear questions, or questions that were not relevant to the local context and made the appropriate adjustments.

### Study design and data collection

From April 22nd, 2018, to May 1st, 2018, we retrospectively reviewed the medical records of surgical and obstetric patients who were diagnosed with SSI and sepsis during the three-month baseline period. During the two weeks, data collectors searched independently for patient files in the medical records departments and surgical wards, and then a member of the research team and a data collector from each of the facilities searched together on a designated chart review day. At this point, if the patient files were not found, then it was considered ‘not found.’ The level completeness of the medical records was assessed by evaluating the rates of 1) documentation of SSI or sepsis and their respective symptoms and vitals, 2) patient daily monitoring and progress information, and 3) documentation of key patient demographics. A ‘complete’ medical record included all of these three components. To operationalize documentation of SSI, we defined SSI as ‘pus draining from the wound,’ ‘closed wound opened,’ ‘wound with a foul smell,’ and ‘wound infection [19].’ Since post-operative complication documentation mechanisms are not uniform across region or health facility, we developed separate criteria to assess documentation of these three components in medical records and, thus, completeness of a medical record (Table 1).

**Table 1.** Criteria developed to assess the completeness of medical records.

Documentation of SSI/sepsis diagnosis
Documentation of SSI: The clinician’s notes include any combination of the following keywords ‘pus draining from the wound,’ ‘closed wound opened,’ ‘wound with a foul smell,’ and ‘wound infection’ ^1^ AND, 1 or more of the following symptoms: heat, redness, localized tenderness, purulent drainage, spontaneous dehiscence, operative findings indicative of infection. Documentation of sepsis: The clinician’s notes include a keyword ‘sepsis’ AND 2 or more of the following criteria: temperature, heart rate, systolic blood pressure, and respiratory rate. We evaluated the presence of recording, not accuracy of measurement.
Patient progress and monitoring
Documentation of the following patient progress and monitoring information: daily progress notes documented for each inpatient day*, doctors’ orders documented on each inpatient day, partograph present and completed during the perinatal period, discharge details documented (discharge, referral, or death date); post-operative notes documented*, indication for cesarean section documented.
Patient characteristics
Documentation of the following patient characteristics: patient history (obstetric admission sheet for obstetric patients), name, age, sex.

*Daily progress notes documented for each inpatient day were written by assistant medical officers/surgeons or another clinical person. Post-operative notes were written by the surgeon directly after the operation.

To examine the level of completion of medical records, we developed a Data Quality Assessment (DQA) tool which included 18 questions addressing three main areas: 1) documentation of SSI, sepsis and their respective symptoms and vitals (Questions: 14, 15, 16), 2) patient daily monitoring and progress (Questions: 5, 6,7, 11, 12), and 3) patient characteristics (Questions: 3, 4). (Appendix II). The tool was developed by the research team and was informed by the World Health Organization (WHO) Western Pacific Region’s Medical Records Manual: A Guide for Developing Countries [20]. Development of the tool was also informed by 5 or 6 semi-structured interviews with key stakeholders (i.e. surgical providers, chief medical officers, surgical nurses, and data managers) by members of the research team to understand data flow from the facility to the national level.

### Data analysis

Data for all found medical records were collected in Excel (Microsoft, Redmond, WA, USA) and were analyzed in Stata/IC 15.1 (College Station, TX, USA). Descriptive analyses were conducted to describe the demographic characteristics of the study population and to summarize medical record completeness rates.

## Results

***Demographics***: During the three-month baseline period, 7%, 6%, and 2% of all post-operative and post-partum patients followed in the 10 intervention facilities (N = 4,343) developed an SSI, post-operative sepsis and maternal sepsis respectively. The procedure most frequently leading to a complication was a cesarean section, with 69% and 53% leading to an SSI and sepsis, respectively (Table A1/A2). A little over one-third of all patients diagnosed with an SSI were in-hospital for over 10 days (34%), while this rate was slightly lower for those diagnosed with sepsis (31%) Table 1A/2A. SSIs and sepsis were diagnosed 6.4 and 4.0 days after hospitalization on average, respectively. Most cases of SSI were superficial (70%) (Appendix III). Of the 157 cases of SSI and sepsis diagnosed, 107 (68%) of the medical records could be located and reviewed. Table 2 shows the demographic profile of all patients who were diagnosed with SSI and sepsis. Only sex and mortality were statistically significantly different (p < 0.05) between patients whose medical records were found, and those whose records were missing (Table 2). Among medical records found, the majority of patients were female (91%) and between the ages of 13–45 years (92%) (Table 2). Approximately two-thirds of the medical records that were found were of patients admitted in district hospitals (63%). One-third of the patients stayed at the health facility for more than 11 days (34%). Patient diagnoses were evenly distributed between SSIs (45%) and sepsis (post-operative or maternal sepsis), with 14% diagnosed with both. Very few medical records were of patients who died at the facility (3%). Most of the medical records reviewed were of surgical patients (85%), while 15% were of women who delivered vaginally (Table 2).

**Table 2.** Patient demographics among found and not found.

Characteristics	Total (n = 157)	Found (n = 107)	Not found (n = 50)	p-value
Sex				
Female	136 (87%)	97 (91%)	39 (78%)	0.030*
Age				
≤ 12 years	4 (3%)	2 (2%)	2 (4%)	0.085
13–45 years	138 (88%)	98 (92%)	40 (80%)	
> 46 years	15 (10%)	7 (7%)	8 (16%)	
Facility Level				
Regional Referral Hospital	43 (27%)	27 (25%)	16 (32%)	0.580
District Hospital	94 (60%)	67 (63%)	27 (54%)	
Health Center	20 (13%)	13 (12%)	7 (14%)	
Length of Stay				
≤ 3 days	24 (15%)	18 (17%)	6 (12%)	0.070
4–10 days	68 (43%)	49 (46%)	19 (38%)	
> 11 days	53 (34%)	36 (34%)	17 (34%)	
Not applicable (died or referred)	12 (8%)	4 (4%)	8 (16%)	
Event Type				
Only SSI	69 (44%)	48 (45%)	22 (44%)	0.280
Only Sepsis	63 (40%)	44 (41%)	17 (34%)	
Both SSI and Sepsis	25 (16%)	15 (14%)	11 (22%)	
Mortality	12 (8%)	3 (3%)	9 (18%)	0.002*
Normal spontaneous vaginal delivery	23 (15%)	16 (15%)	7 (14%)	1.000

Percentages may not add to 100% due to rounding.

*p-value <.05.

*Completeness of perioperative documentation*: Among surgical patient medical records, 87% included post-operative notes, and among cesarean section patient medical records, 87% included an indication for a cesarean section (Table 3).

**Table 3.** Completeness of perioperative documentation (among medical records found).

	Post-operative notes n (%) (n = 91)	Indication of cesarean section n (%) (n = 70)
Completed	79 (87%)	61 (87%)

*Documentation of SSI and sepsis diagnosis and associated symptoms and vitals*: Among the medical records reviewed with SSI cases, a third (34%) of the medical records included documentation of a diagnosis of SSI and 57% documented signs or symptoms of SSI. 23% of the medical records with sepsis cases documented a diagnosis of sepsis in the medical records, and two-thirds (62%) documented two or more vital signs of sepsis (Table 4).

**Table 4.** Documentation of SSI and sepsis, and recorded symptoms and vitals (among medical records found).

	SSI n (%) (n = 61)	Sepsis n (%) (n = 60)
Documentation of diagnosis	21 (34%)	14 (23%)
Documentation of symptoms or/and vitals*	35 (57%)	37 (62%)
No Documentation at all	5 (8%)	9 (15%)

*Documentation of symptoms or/and vitals also have a documentation of diagnosis.

*Completeness and utilization of inpatient clinical progress information*: Among medical records found, the majority (76%) included patients’ history. Less than half (41%) of the daily progress notes were written on all days of admission, and less than one-third (29%) medical records a daily record of doctor’s orders. Among obstetric patient records, 79% included a partogram (Table 5).

**Table 5.** Completeness and utilization of inpatient clinical progress data/information? (among medical records found).

	n (%) (n = 107)
Patient history included	81 (76%)
Daily progress notes written	44 (41%)
Doctors’ orders documented	31 (29%)
Partogram Utilized*	69 (79%)

*Among obstetric patients (n = 87).

*Overall completeness of medical records*: 6% of medical records were complete with documented SSI and sepsis diagnosis, vitals, inpatient clinical progress perioperative documentation indicators, as defined by the research team. We also examined the completeness of medical records among surgical and obstetric patients, and documentation rates were similar (Table 6)

**Table 6.** Overall completeness of medical records (among medial records found).

	All patients n (%) (n = 107)	Surgical patients n (%) (n = 92)	SVD patients n (%) (n = 16)	Cesarean section patients n (%) (n = 70)
Completeness of medical records	6 (6%)	5 (5%)	1 (6%)	4 (6%)

1. Forrester JA, Koritsanszky LA, Amenu D Developing Process Maps as a Tool for a Surgical Infection Prevention Quality Improvement Initiative in Resource-Constrained Settings. *J Am Coll Surg*. 2018;226(6):1103–1116.e3. doi:10.1016/j.jamcollsurg.2018.03.020.

## Discussion

Our results show that 32% of the medical records of patients diagnosed with SSI and sepsis were not present at the time of the assessment. Besides missing medical records these findings show that primary data quality is weak in health facilities in northern Tanzania. This is consistent with previous studies examining HMIS in Tanzania at the national level that have also found data quality and reporting to be inefficient and ineffective at the district and national levels [11,13]. Unsatisfactory MTUHA register completion for non-surgical indicators implies that large-scale public health decisions are informed by rough aggregate estimates based on incomplete data [13]. Our study further highlights this concern by demonstrating that in addition to problems in reporting at the district and national levels, health facilities struggle with collecting, storing, and reporting of patient data at the facility level, which is essential for improving quality of surgical care. Nevertheless, as previous research demonstrates, data quality improvement is a slow process and necessitates a significant change in organizational culture and processes [21].

Despite ongoing efforts, implementation of Electronic Medical Record systems in Tanzania are limited and fragmented, and most facilities depend on paper-based recording of facility-level data [22]. As long as facilities continue to depend on paper records, safe storage and management of patient files are essential. SSIs and sepsis typically develop 5–10 days postoperatively, usually after the patient is discharged from the facility [23–26]. Studies in high-income countries, as well as LMICs, have found that SSIs are a leading cause of hospital readmissions [27,28]. Thus, timely access to the medical records of returning patients ensures continuity of care at readmission and improves quality patient outcomes. Anecdotally, we found that the reasons for missing medical records were due to inconsistencies in using unique patient identifiers and a lack of storage space for medical records, which is reflective of a lack of infrastructure and human resources to effectively manage medical records.

We found that most facilities do not explicitly document a diagnosis of SSI and sepsis. While documentation rates of vital signs and symptoms are higher, they are still inadequate. Both the explicit documentation of SSI and sepsis, as well as vital signs and symptoms, are essential for improved tracking, diagnosis, and treatment of SSI and sepsis at the facility level. The Ministry of Health of Tanzania mandated that MTUHA be comprehensive in scope. However, thus far, MTUHA has primarily focused on maternal and child health indicators [11]. The absence of mandated surgical indicator reporting on the national level may lead to a lack of accountability at the facility level. One way to improve accountability could be the inclusion of surgical indicators in the MTUHA system to encourage accurate and timely collection, aggregation, and reporting of these indicators to the national level. Improving accurate documentation of surgical indicators is key to effective surveillance, providing facilities with an accurate picture of baseline post-operative complication rates and the existing gaps. Documentation alone does not ensure data accuracy; however, it might improve patient outcomes and is thus the first step towards improving quality of care [29].

Analysis of the completeness and utilization of inpatient clinical progress indicators such as patient history, daily progress notes, doctors’ orders, and partogram shows that patient history and partograms were completed and used in most medical records. However, daily progress notes and doctors’ orders were not written regularly. This could be due to our conservative measure of completeness for daily progress notes and doctors’ orders, which had to be completed daily, for the duration of the patient stay at the facility. We acknowledge stringent data recording processes should not be an end in themselves but instead used to monitor performance, identify gaps in care, provide feedback with the ultimate goal of improve quality of care. However, regular post-operative monitoring is essential to identify early signs and symptoms of SSIs and prevention of surgical complications [30]. Lack of monitoring and documentation of patient progress might lead to patients being discharged too early and without adequate care. Although facilities faced challenges in recording inpatient clinical progress indicators, most medical records included post-operative notes and an indication for cesarean section. Completeness of post-operative notes is crucial for the continuity of care, especially in settings where task-shifting is the primary mode of care delivery, and doctors use post-operative notes to outline treatment plans.

There are a few limitations to this study. Due to the limited resources and difficulty obtaining medical records from the records department, we were unable to retrospectively review the quality of all medical records in our study. The infrastructural capacity varied between the facilities, ranging from well-staffed and equipped medical records departments to those with facilities lacking space and human resources. For this reason, our study sample consisted of patients who were diagnosed with SSI and sepsis by the medical data collectors as part of the baseline data collection for the study because they were high-risk patients rather than healthy patients whose medical records might be lost due to their short inpatient stay and lack of follow-up. In addition, since the assessment was conducted towards the end of baseline data collection, there is a possibility of a Hawthorne effect since the data collectors were present and observing data collection practices, which might have impacted the surgical teams’ behavior.

Finally, the assessment mainly focused on one element of data quality – completeness. Other aspects of data quality, including accuracy and reliability, were not evaluated. Tracking data accuracy would require real-time observation of data recording. In our study design, we accounted for a retrospective medical record review as the most cost-effective and efficient means to evaluate data quality.

## Conclusion

Quality improvement for safe surgical care is contingent on robust data and consistent record-keeping practices at the facility level. Accurate and reliable medical records provide patient information that can be used to improve continuity of care, communication, and coordination among the surgical providers and ancillary teams as well as hospital managers. Accurate medical records also provide data for decision making for facility-level quality improvement. Furthermore, appropriate reporting of facility-level indicators helps inform evidence-based decision making at the regional and national levels.

Given the far-reaching impact of the quality of medical records for surgical and obstetric patient care, our findings highlight the need to address gaps in primary data quality. Previously implemented strategies to strengthen routine data quality include periodic data quality checks by district MOH staff, rapid feedback to resolve data gaps, and implementation of simple report cards/data dashboards to provide aggregate comparisons of key indicators [31]. Job aids and supervision tools have also been utilized to improve health facility data collection and use [31]. These methods, which have been tested in low-resource settings, could be adapted to the local context and used to improve data completeness in Tanzanian health facilities. Robust record keeping is essential for patient safety and continuity of care, communication between the hospital staff, and the overall quality of care. Surgical quality is multifaceted and notoriously difficult to measure, which further speaks to the importance of accurate surveillance of SSI and sepsis as indicators of surgical quality. The ability to successfully record surgical data is the first step towards measuring and improving quality.

## Paper context

Reliable and complete patient data are essential for surgical quality improvement in low-resource settings. However, the evidence is sparse on the state of paper-based record collection. This study shows significant gaps in medical record availability and completeness for surgical patients diagnosed with surgical site infections and sepsis. Strategies to strengthen routine data quality such as data quality checks, usage of simple report cards, and rapid feedback for process improvement could fill some of these gaps.

## Appendix I. Minor Surgeries excluded from the study

Minor surgeries excluded from the study included:

- Excisional biopsy of skin and soft tissue
- Suturing and laceration
- Evacuation of products of conception
- Suture removal
- Dressing changes
- Exam under anaesthesia
- Foreign body removal
- Incision and drainage of superficial abscess
- Tooth extraction
- Closed reduction of fractures
- Plaster case application
- Chest tube replacement
- Catheter change
- Minor burn dressing

## Appendix II.

Data Quality Assessment used in the study Patient Characteristics

1. Region [Insert]
2. Facility Name [Insert]
3. Specific diagnosis (Maternal sepsis/surgical site infection/post-op sepsis)
4. PGSSC study ID [insert]
5. Patient age [insert]
6. Sex (M/F)
7. Type of procedure [insert]
8. Admission date [insert]
9. Discharge date [insert]
10. Readmits (Yes/No/Not applicable)
11. Death (Yes/No/Not applicable)

Patient file assessment

1. Patient file present (Yes/No)
2. Is the patient file physically intact? (Yes/No)
3. Are demographic details included specifically name, age, and sex (Yes/No/Not applicable)
4. Is patient history included? Please note for obstetric patients this would be the obstetric admission sheet that is used (Yes/No/Not applicable)
5. Were daily progress notes written on all days of admission? (Yes/No/Not applicable)
6. Were operative notes written with operative findings and diagnosis included? (Yes/No/Not applicable)
7. Are the doctor’s orders documented everyday patient was in the ward? (Yes/No/Not applicable)
8. Was ASA class documented correctly in the OR logbook? Please note this will be compared with either the external data collectors’ tool or their clinical assessment of the patient (Yes/No/Not applicable)
9. Was wound class documented correctly in OR logbook? Please note this will be compared with either the external data collectors’ tool or their clinical assessment of the patient (Yes/No/Not applicable)
10. Was the partogram utilized? (Yes/No/Not applicable)
11. Was the indication for C-section documented? (Yes/No/Not applicable)
12. Discharge details present? (Yes/No/Not applicable)
13. Was there a specific mention of SSI or wound infection in the patient file? (Yes/No/Not applicable)
14. Was 1 or more diagnostic criteria for SSI recorded – this includes heat, redness, localized tenderness, purulent drainage, spontaneous dehiscence, operative findings indicative of infection? (Yes/No/Not applicable)
15. Was there specific mention of sepsis? (Yes/No/Not applicable)
16. Which vitals were documented:

a. Was altered mental state recorded? (Yes/No/Not applicable)

b. Was temperature recorded? (Yes/No/Not applicable)

c. Was the heart rate recorded? (Yes/No/Not applicable)

d. Was the BP recorded? (Yes/No/Not applicable)

e. Was the respiratory rate recorded? (Yes/No/Not applicable)

1. Was vaginal tear/episiotomy documented? (Yes/No/Not applicable)
2. Any discrepancies between patient file and external data collector? [insert]

## Appendix III. Summary Tables

**Table A1.** Characteristics of patients diagnosed with an SSI.

	Diagnosed with an SSI (n = 93)	
	n	%
Sex		
Female	78	83.9%
Age		
≤ 12 years	3	3.2%
13–45 years	13	82.8%
> 46 years	77	14.0%
Procedure type		
Cesarean section	64	68.8%
Exploratory laparotomy	17	18.3%
Hernia repair	0	0.0%
Prostatectomy	6	6.5%
Hysterectomy	1	1.1%
Appendectomy	1	1.1%
Other	4	4.3%
Length of stay		
≤ 3 days	5	5.4%
4–10 days	32	60.2%
>10 days	56	34.4%
Average time until diagnosis (days)	6.4	-
Pre-operative antibiotic administration rate*	38	40.9%
Post-operative administration rate	92	98.9%
Type of SSI		
Superficial	65	69.9%
Deep	22	23.7%
Organ space	6	6.5%

*n = 2 missing

**Table A2.** Characteristics of patients diagnosed with sepsis (maternal or postoperative).

	Diagnosed with sepsis or maternal sepsis (n = 88)	
Characteristics	n	%
Sex		
Female	77	87.5%
Age		
≤ 12 12 years	2	2.3%
13–45 years	81	92.0%
> 46 years	5	5.7%
Procedure type		
Cesarean section	47	53.4%
Normal spontaneous vaginal delivery	22	25.0%
Exploratory laparotomy	11	12.5%
Hernia repair	3	3.4%
Prostatectomy	1	1.1%
Hysterectomy	1	1.1%
Appendectomy	0	0.0%
Other	3	3.4%
Length of stay		
≤ 3 days	13	14.8%
4–10 days	48	54.5%
>10 days	27	30.7%
Average time diagnosis (days)	4.0	-
Pre-operative antibiotic administration rate*	39	59.1%
Post-operative administration rate*	62	93.9%
Type of sepsis		
Severe sepsis	9	10.2%
Septic shock	1	1.1%
No severe sepsis or septic shock	78	88.6%

*The denominator includes n = 66 surgical patients

1. Forrester JA, Koritsanszky LA, Amenu D Developing Process Maps as a Tool for a Surgical Infection Prevention Quality Improvement Initiative in Resource-Constrained Settings. *J Am Coll Surg*. 2018;226(6):1103–1116.e3. doi:10.1016/j.jamcollsurg.2018.03.020
